# Assessing the Limit of CO_2_ Storage in Seawater as Bicarbonate-Enriched Solutions

**DOI:** 10.3390/molecules29174069

**Published:** 2024-08-28

**Authors:** Selene Varliero, Samira Jamali Alamooti, Francesco Pietro Campo, Giovanni Cappello, Stefano Cappello, Stefano Caserini, Federico Comazzi, Piero Macchi, Guido Raos

**Affiliations:** 1 Department of Chemistry, Materials and Chemical Engineering “Giulio Natta”, Politecnico di Milano, Via Luigi Mancinelli 7, 20131 Milano, Italy; 2 Limenet®, Via Giovanni Amendola 4-6, 23900 Lecco, Italy; 3 Department of Civil and Environmental Engineering, Politecnico di Milano, Piazza Leonardo Da Vinci 32, 20133 Milano, Italy; 4 Department of Engineering and Architecture, Università di Parma, Parco Area delle Scienze 59, 43124 Parma, Italy

**Keywords:** CO_2_ storage, climate change mitigation, marine chemistry, solution equilibria, carbonate system

## Abstract

The dissolution of CO_2_ in seawater in the form of bicarbonate ions is an attractive alternative to storage in geological formations, on the condition that the storage is stable over long periods and does not harm the marine environment. In this work, we focus on the long-term chemical stability of CO_2_ absorbed in seawater as bicarbonate by monitoring the physico-chemical properties of the solutions (pH, dissolved inorganic carbon and alkalinity) in six different sets of experiments on both natural and artificial seawater lasting up to three months. The bicarbonate treatment of natural seawater consists of mixing it with pre-equilibrated solutions obtained from the reaction of CO_2_ and Ca(OH)_2_, with the same pH as natural seawater. This was achieved with a pilot plant working with tons of seawater, while small-scale laboratory experiments were carried out by adding sodium bicarbonate to artificial seawater solutions. If the increase in the overall carbon concentration in the final mixture does not exceed a critical threshold (about 1000–1500 μmol/L), the resulting bicarbonate-rich solutions are found to be stable for over three months.

## 1. Introduction

The permanent storage of carbon dioxide (CO_2_) is vital in virtually all mitigation scenarios compatible with ambitious climate targets. CO_2_ storage could be used both for the CO_2_ captured from the flue gas of industrial processes and for the CO_2_ sequestered from the atmosphere through artificial processes [1].

The most developed approach for storing CO_2_ is geological storage, namely the injection of CO_2_ into geological formations, e.g., in deep saline aquifers [2]. Because the pace and scaling of geological CO_2_ storage deployment have fallen short of expectations, and considering that this approach is unfeasible in many geographical areas [3,4,5], there is increasing interest in alternative solutions that could provide permanent storage of large quantities of CO_2_.

Many authors have proposed and studied the storage of carbon dioxide in seawater [6,7,8,9,10], which already contains 98% of the overall CO_2_ in the combined ocean-atmosphere system [11]. The large majority of this (86.5%, on average) is actually in the form of bicarbonate ions (HCO_3_^−^) [11]. Marine storage of CO_2_ in the form of bicarbonate ions has the potential to last for geologic times, on the order of 10,000 years [12,13,14]. Rau and Caldeira [6,15] proposed a method called Accelerated Weathering of Limestone (AWL), consisting of the reaction of CO_2_ from power plants’ exhaust gas with seawater and calcium carbonate minerals (CaCO_3_), namely calcite or aragonite, with a final discharge into the ocean of an ionic solution rich in bicarbonates. The overall “weathering” reaction may be summarized as follows:

CaCO_3_(s) + CO_2_(g) + H_2_O(l) → Ca^2+^(aq) + 2HCO_3_^−^(aq) (1)

This method has progressed from the laboratory level [16] to a feasibility case study [17], to a pilot-scale reactor [18], and to modeling of local impacts on seawater carbonate chemistry [19]. An improvement of this method, named buffered accelerated weathering of limestone (BAWL), has been proposed by Caserini et al. [9]. With this approach, CO_2_ is used in stoichiometric excess with respect to the carbonate minerals, but calcium hydroxide [Ca(OH)_2_, also known as slaked lime, SL] is added in the final stages of the process to produce a buffered ionic solution at the same pH as seawater. De Marco et al. [20] investigated mass and energy balances and the costs of applying BAWL to the capture and storage of CO_2_ from the flue gas of an existing industrial source, and concluded that the process is technically feasible and economically viable.

One intrinsic shortcoming of the AWL and BAWL is the slow rate of the reaction between aqueous CO_2_ and limestone. As a consequence, big plants treating large amounts of seawater would be necessary for marine storage of CO_2_. The process implemented by Limenet^®^ company [21] is an evolution of BAWL that attempts to overcome this problem by the direct combination of CO_2_ with Ca(OH)_2_, to induce the overall reaction:

2CO_2_(aq) + Ca(OH)_2_(s) + H_2_O(l) → Ca^2+^(aq) + 2HCO_3_^−^(aq) (2)

The reaction is carried out in specially designed reactors, where CO_2_ is first dissolved in seawater, and then Ca(OH)_2_ is added to give a bicarbonate-enriched solution with a pH equal to that of natural seawater. As an additional benefit, the solution has high alkalinity, thus increasing the buffering capacity of seawater against acidification [22]. For this reason, these technologies are classified as Ocean Alkalinity Enhancement (OAE) processes. The SL employed in reaction (2) is typically produced by calcination of limestone, an energy-intensive process that produces one mol of CO_2_ per mol of CaCO_3_. Any additional CO_2_ emissions can be avoided by using renewable energies for the calcination and by sequestering the CO_2_ with one-half of the produced Ca(OH)_2_. Therefore, ideally this process enables the net sequestration of one mol of CO_2_ per mol of CaCO_3_. Several recent scientific studies address the possible beneficial or harmful consequences of OAE implementations on marine biota [23,24], and the future efficiency of its large-scale implementation [25,26].

The fundamental question that inspired this research is whether the increased amount of bicarbonate in seawater remains stable over time and, therefore, fulfills the requirements for permanent storage. The aim is also to identify the optimal relative amounts of seawater, CO_2_, and Ca(OH)_2_ that avoid CO_2_ degassing as well as abiotic or biotic precipitation of carbonate minerals. These are two of the strongest pitfalls of such approaches, as carbonate precipitation would lead to the re-emission of CO_2_ into the atmosphere by a reaction that is essentially the reverse of (1):

Ca^2+^(aq) + 2HCO_3_^−^(aq) → CaCO_3_(s) + CO_2_(g) + H_2_O(l). (3)

These questions were prompted, among other things, by analogous studies of the stability of seawater treated by ocean liming (OL) operations [7,8,12]. OL consists of the direct dispersion of Ca(OH)_2_ on the surface of seawater to induce additional absorption of atmospheric CO_2_ [12]. Those studies demonstrated that, apart from causing potentially harmful spikes in seawater pH, such OAE operations may also be ineffective because they can trigger unwanted side reactions like (3). While classical ocean liming is an unequilibrated process, the injection of a bicarbonate solution has the inherent advantage of leaving the seawater pH unaltered. In fact, the dissolution of calcium hydroxide occurs in a closed system and with the exact amount of water needed. Only afterward is the bicarbonate-enriched marine solution released into the sea at the same pH. This pH-equilibrated marine solution implies fewer serendipities and unpredictable behaviors than ocean liming, especially pH spikes and possible precipitation of carbonates. By pH-equilibrated, we indicate a solution with the same pH as natural seawater.

Equilibrium with respect to pH and any other chemical reaction within the aqueous phase does not automatically imply equilibrium with respect to other phases, including the formation or dissolution of minerals and the uptake or release of gaseous atmospheric CO_2_ [11]. In this respect, it is important to stress that the ocean surface is heavily supersaturated in carbonate minerals, implying a high risk of precipitation. A sudden and uncontrolled increase in the local concentration of carbonate ions may trigger the nucleation and therefore the precipitation of carbonate minerals. In particular, the aragonite saturation state ranges between 2.7 and 3.7 in the Mediterranean Sea [27]. It is defined and calculated by the following equation:

(4) $$\Omega_{Ar}=\frac{\left[Ca^{2+}\right]\left[CO_{3}^{2-}\right]}{K_{SP}}$$

where $\left[Ca^{2+}\right]$ and $\left[CO_{3}^{2-}\right]$ are the molar concentrations of calcium and carbonate ions, while *K_SP_* is the stoichiometric solubility product of aragonite in seawater [27]. The aragonite saturation state is considered a useful indicator of the risk of precipitation [7,28,29], even though it is more soluble than calcite, as precipitation of the latter is inhibited by the high concentration of magnesium in seawater [30].

This work aims to assess the storage efficiency of CO_2_, converted into bicarbonate ions, in seawater. In particular, it is important to quantify the limit of bicarbonate additions without causing side effects such as the precipitation of carbonate minerals (e.g., aragonite or calcite) that may occur several days or even weeks after treatment. With this in mind, we have tested the stability of seawater solutions containing an enhanced concentration of bicarbonate ions in two distinct sets of experiments:

(a) Natural seawater treated with the Limenet^®^ process at a site located in the harbor of La Spezia (Italy) and subsequently transferred to our laboratory at the Politecnico di Milano for long-term monitoring;
(b) Artificial seawater prepared and treated in the laboratory with controlled additions of sodium bicarbonate.

Furthermore, we have evaluated the durability of CO_2_ stored in the form of dissolved bicarbonates through measurements of pH, Dissolved Inorganic Carbon (DIC), and Total Alkalinity (TA).

Within the scope of our study, it is important to stress that DIC approximately coincides with the sum of C contained in HCO_3_^−^ and CO_3_^2−^ because the smaller contribution of CO_2_ can be ignored in seawater, and no other inorganic C is present. On the other hand, TA is approximately the sum of the quantities of HCO_3_^−^ and 2 times CO_3_^2−^. Both indicators are therefore useful for monitoring the C content in seawater. Our observations have been correlated with the calculated saturation states (Ω) of calcite and aragonite [Equation (4)]. We monitored these parameters over long periods, ranging from a few days up to three months, allowing us to assess the stability of the treated solutions.

## 2. Results

Table 1 summarizes the series of experiments we conducted to test the stability of bicarbonate-enriched seawater solutions. The first column contains labels used throughout the manuscript to indicate a series of samples and experimental conditions. These can be classified according to the following variables (see Section 4 for more details):

(1) Mode: Carbon was added to the solutions either in a single step or by multiple additions over a period of several days.
(2) Seawater: We used either natural seawater (collected from the Mediterranean Sea at La Spezia) or artificial seawater (prepared from purified water and inorganic salts).
(3) Environment: We measured the evolution of the treated solutions either in an open atmosphere or in closed cabinets with a fixed volume of enclosed air (ca. 300 L). We call the experiments as “mixed” where we temporarily opened the cabinet to perform the addition of sodium bicarbonate.
(4) Treatment: The alkalinization of seawater was obtained either with a concentrated solution of sodium bicarbonate or through the Limenet^®^ process. The latter implies the formation of calcium bicarbonate from the neutralization of carbon dioxide and calcium hydroxide, as described in the Introduction and in Section 4. These treatments are indicated in the table as NaHCO_3_ and Ca(HCO_3_)_2_, respectively.
(5) MaxΔ_DIC_: The largest theoretical amount of added carbon (in µmol/L) for a series of experiments. It is a theoretical value because it represents the expected increase in DIC, assuming ideal addition without degassing or precipitation.
(6) Initial DIC: In the experiments with natural seawater, the measured initial DIC was 2370 µmol/L for SN1/SN2 and 2470 µmol/L for MN. In the experiments with artificial seawater (MA and SA), the initial DIC was set to 2000 µmol/L [31] or to 2800 µmol/L, obtained from the dissolution of NaHCO_3_.
(7) Duration: This refers to the longest duration of a set of experiments. Measurements were carried out in the laboratory for up to 90 days.

**Figure 1:**
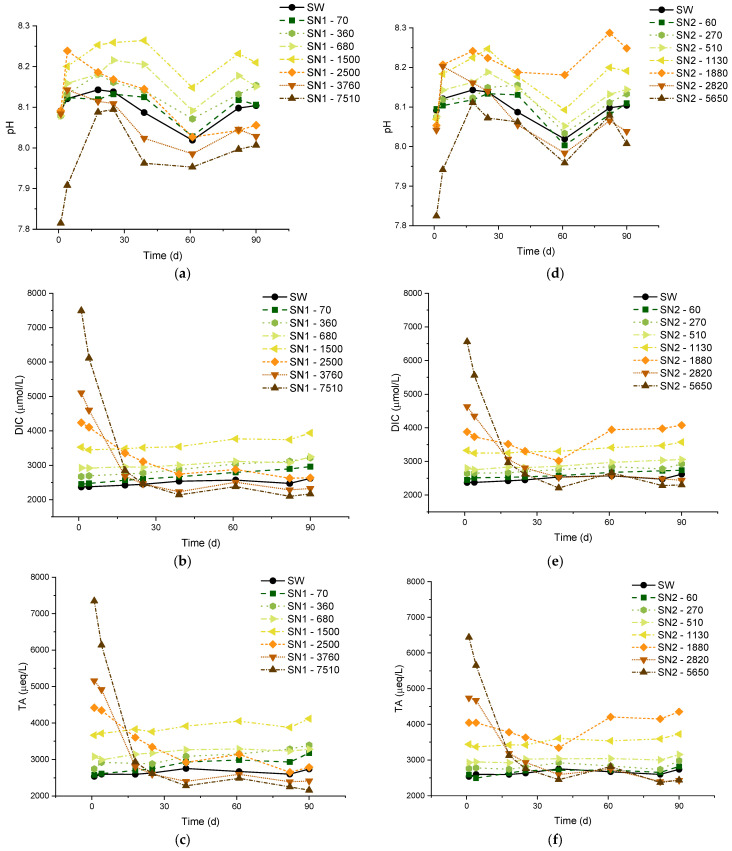
Measured pH, alkalinity, and DIC values over 90 days. Graphs (**a**–**c**) refer to SN1, and graphs (**d**–**f**) refer to SN2.

In Figure 1, we report results from the experiments of types SN1 and SN2, which are characterized by different values of MaxΔ_DIC_. The measurements lasted up to 90 days, which is one of the longest periods ever reported in the literature for this type of study. The numbers next to each code (e.g., 70 in “SN1-70”) indicate the theoretical added DIC, in µmol/L. We measured the pH, DIC, and TA with variable frequency. We also report the results of concomitant control experiments on untreated natural seawater (SW) used as a reference. The average starting pH of the SW samples we analyzed is ca. 8.1, close to the values reported in the literature for the Mediterranean [27]. We point out that the solutions monitored in SN1 and SN2 experiments were static, as we did not continuously stir or vibrate them to mimic the natural motion of the ocean surface. Some stirring was nonetheless applied almost daily, at least in the initial phases of the experiments, as part of the sampling operations.

A few minutes after the initial dissolution (“day 0”), all the samples share the same pH as SW, apart from the two solutions with the highest Δ_DIC_ (7510 μmol/L for SN1 and 5650 μmol/L for SN2), which have a lower pH. This is probably caused by partial precipitation of carbonate minerals occurring in the initial stages of the treatment, before the first pH measurement. Nonetheless, even in these two solutions, the pH increases until day 18, when the gap with the other solutions is greatly reduced, even though it remains below that of SW. The pH of the solutions with an added DIC below 270 μmol/L does not show a systematic trend compared to SW, although the differences with respect to SW are always below 0.04, well within the precision limits of the measurements. This behavior indicates that pH is not significantly affected by low DIC additions. For solutions with carbon addition between 360 and 1500 μmol/L, the pH is consistently higher than in SW, proportional to the theoretical concentration. Note that a small increase in pH is expected to be beneficial for the marine environment, considering that the oceans have already undergone significant acidification (the average pH has decreased from 8.11 in 1985 to 8.05 in 2021) due to the enhanced absorption of CO_2_ from the atmosphere [32], and that a surface ocean pH as low as recent times is uncommon in the last two million years [33].

The rest of Figure 1 reports results for the DIC [panels (b) and (e)] and the TA [panels (c) and (f)]. The measurements of these quantities started on “day 1”, immediately after the arrival of the seawater samples at our laboratory. The overall behavior of these quantities is consistent with our pH measurements. In both the SN1 and SN2 series of experiments, the two solutions treated with the largest additions show a decrease in DIC and TA to levels lower than in SW within approximately 30 days. Note that, for most of the samples, the measurements of DIC indicate values already lower than the sum of the initial DIC and the theoretical Δ_DIC_ (see again Table 1). This suggests the occurrence of some precipitation and degassing for high DIC additions, which will be taken into account in the formulation of the process efficiency, below. On the other hand, untreated SW and the solutions with Δ_DIC_ equal to 1500 µmol/L or lower show a slight increase in TA and DIC for the entire duration of the monitoring.

The precipitation of carbonates from the most concentrated solutions is not surprising, considering the natural supersaturation of seawater [27]. The saturation states Ω of all solutions under examination can be computed from the measured pH, TA, and DIC values [11], and they show some variation. We should consider that the samples were not stored in a temperature-controlled ambient; therefore, the Ω of untreated natural seawater also fluctuated during the control period: the initial Ω was 6.45 and 4.20 for calcite and aragonite, respectively, and the two quantities varied in the ranges 5.15–8.84 (calcite) and 3.34–5.69 (aragonite) without the occurrence of precipitation (Table A1 and Table A2 in Appendix A). Of course, analogous oscillations also affected the treated solutions. Therefore, for each measurement, we focus on the saturation of the treated solutions (Ω_i_) relative to the saturation of the control SW measured on the same day (Ω_SW_), using the ratio:

rΩ = Ω_i_/Ω_SW_. (5)

Calcite and aragonite share the same rΩ because the solubility products disappear from the denominators when computing Equation (5).

**Figure 2:**
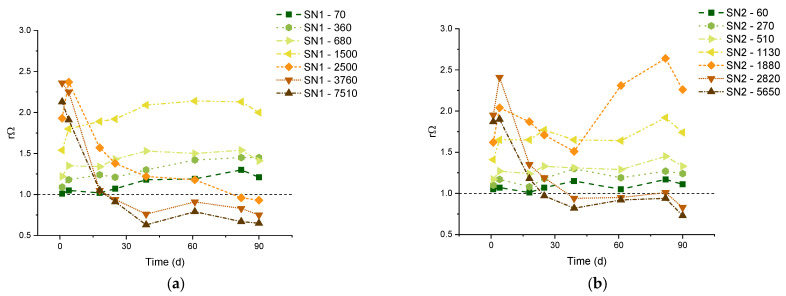
Relative supersaturation rΩ of aragonite and calcite in the SN1 (**a**) and SN2 (**b**) experiments.

The results are reported in Figure 2. The samples with carbon additions of 5650 μmol/L and 2820 μmol/L are those with the largest rΩ on day 1, which rapidly decreases due to observed massive precipitation. The samples with carbon additions of 1500 μmol/L (for the SN1 experiments) and 1130 μmol/L (for the SN2 experiments) have the largest stable rΩ values, respectively equal to 1.94 and 1.68 (average values). So, according to the present study, these rΩ’s could be considered safe threshold values, below which precipitation of carbonate minerals does not occur in our samples.

**Figure 3:**
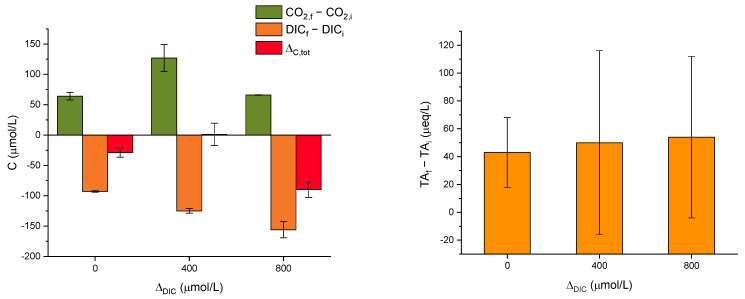
Results of the SA experiments; the error bars refer to individual measurements.

Figure 3 reports results from the SA experiments on artificial seawater with a single addition of alkalinity in the form of NaHCO_3_ powder. Additional data are contained in Table A3 in Appendix A. We tested carbon concentrations of 2000, 2400, and 2800 µmol/L. Each experiment was repeated twice. Considering the value of 2000 µmol/L as a baseline close to untreated natural seawater (see again Table 1), these experiments are labeled as Δ_DIC_ = 0, 400, and 800, respectively. The SA experiments were monitored in a sealed cabinet, which also allowed for the measurement of CO_2_ concentration in the atmosphere. The variation of CO_2_ over time should reflect degassing from the solution.

The pH stabilizes in all the SA experiments, from 7.93 to 8.03. Instead, the TA shows sizeable fluctuations, which are largely due to the technical difficulty of these measurements. As shown in Figure 3, in all three additions, the DIC measured just after the dosage decreases at the end of the experiment to about 100–150 µmol/L, depending on the dosage (values reported as DIC_f_−DIC_i_). This gap increases with the DIC addition, suggesting degassing of CO_2_. This hypothesis is confirmed by the measured increase in atmospheric CO_2_ (CO_2,f_−CO_2,i_), although it is not consistent with the DIC addition. Overall, we can define Δ_C,tot_ as the sum of DIC_f_−DIC_i_ and CO_2,f_−CO_2,i_. We see that all the experiments show a loss of carbon which is not present either in solution or in air. The missing carbon is likely due to minor precipitation of carbonates. We were not able to retrieve the expected quantities in the form of powder after filtration, precisely because these were very small.

Another laboratory experiment (MAM in Table 1) was carried out with eight regular additions, starting again from 2000 µmol/L up to a theoretical DIC of 5200 µmol/L (hence, a Δ_DIC_ of 3200 µmol/L).

**Figure 4:**
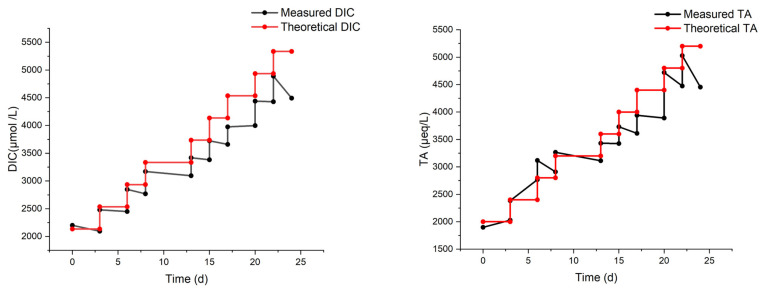
Measured DIC and TA from the MAM experiment on artificial seawater with multiple bi-carbonate dosages over 24 days.

The results of the MAM experiment are reported in Figure 4. The measured DIC increases, but it is progressively lower than the expected value. It is noteworthy that the last addition did not produce any increase in DIC. The total alkalinity, also shown in Figure 4, reflects the same behavior as DIC, though with a pair of outliers on day 6, possibly due to a calibration pitfall. It should be considered, in fact, that the precision of DIC measurement (repeated 3–4 times for each sampling) is much superior to that of TA (single measurement for each sampling).

Even accounting for the lower precision, the drop in TA (compared to the theoretical value) seems to be delayed with respect to the drop in DIC (see again Figure 4). For example, after the third addition on day 8, the TA still matches the theoretical value, while the DIC does not. This may be ascribed to some CO_2_ degassing occurring after the first additions, while the loss of carbon by precipitation (with a concurrent decrease in DIC and TA) would be triggered only subsequently. Indeed, the formation of a few particles was visually observed at two stages of the MAM experiments:

(1) A few days after the third injection of NaHCO_3_ (with a theoretical DIC of 3200 µmol/L), some precipitates floated on the surface of the solution;
(2) At the endpoint of the experiment (theoretical DIC = 5200 µmol/L), a significant number of precipitates stuck on the wall and bottom of the beaker were observed.

The first episode of precipitation occurred during the longest shift at a fixed concentration, enough to allow precipitation. This is the point where the differences between the measured and the theoretical DIC and TA start to increase significantly. Calculated Ω_Ar_ rises from 0.88 (on day 0) to 6.37 (on day 22) and then drops due to precipitation.

**Figure 5:**
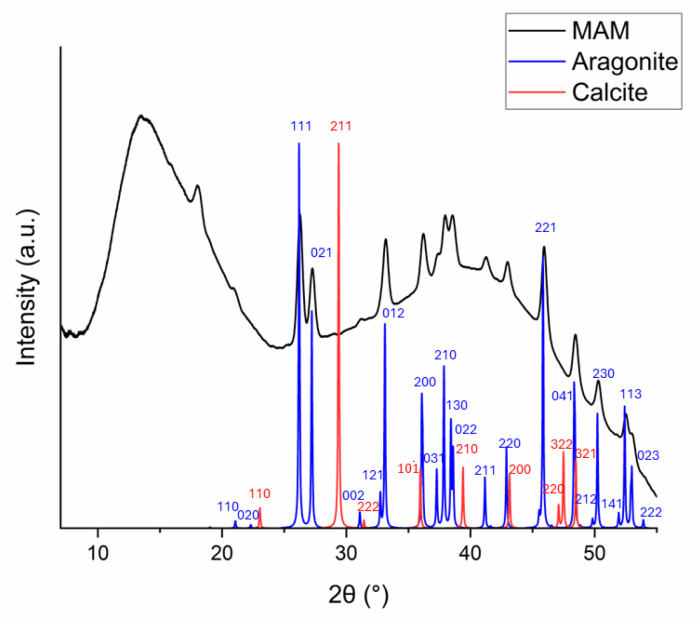
The XRD pattern of the precipitate collected at the end of the MAM experiment (black curve). Simulated diffraction patterns of calcite (red) and aragonite (blue) are also displayed.

The precipitates from the MAM experiment were collected and analyzed by XRD. The diffraction pattern, shown in Figure 5, has clear signatures of the presence of aragonite. The large bump at low diffraction angles is mainly due to scattering from the sample holder and air, while the second one at higher angles is likely due to an amorphous carbonate phase and small precipitation nuclei [30]. From the diffraction pattern it is not possible to recognize any other crystal form than aragonite (and certainly exclude the presence of calcite), despite the fact that aragonite is more soluble (it has a higher K_SP_) than calcite. It is well known that kinetic factors may dominate over thermodynamic ones in the precipitation of carbonates from seawater [34].

**Figure 6:**
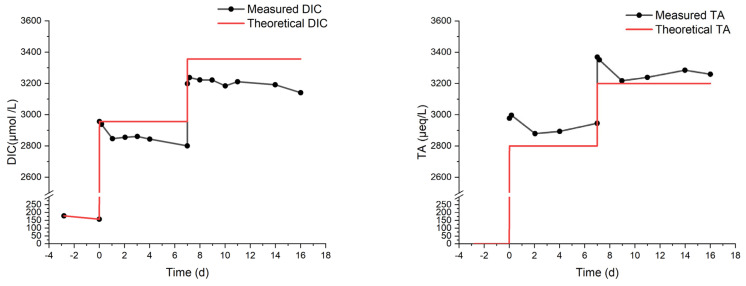
DIC and TA from the MAC experiment in artificial seawater with two-step bicarbonate dosage. Initial TA is assumed to be 0 because it was below the detection limit of the instrument, while for DIC the starting point was measurable by the instrument.

**Figure 7:**
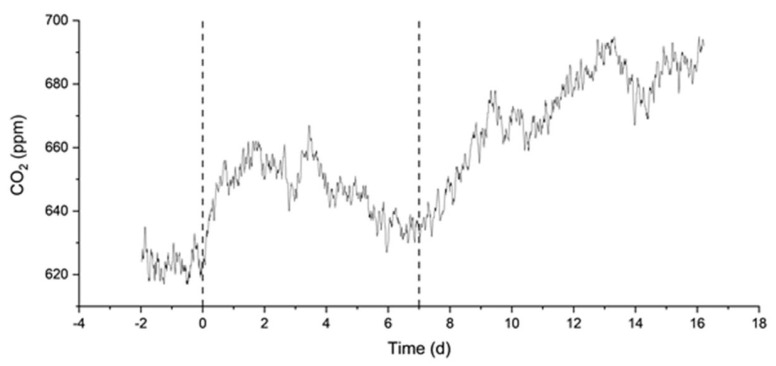
Measured pressure of CO_2_ (in atm) from a MAC experiment in artificial seawater with two-step bicarbonate dosage. Dashed lines indicate the additions of NaHCO_3_ on days 0 and 7.

**Figure 8:**
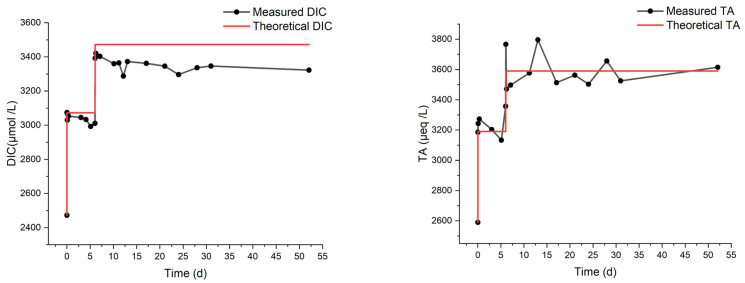
DIC and TA from the MN experiment on natural seawater with two-step bicarbonate dosage.

Finally, we describe the MAC and MN experiments. They were carried out to compare the response of artificial and natural seawater to alkalinity addition. These experiments lasted 16 and 52 days, respectively, with an NaHCO_3_ addition one week after the start of the experiments. The final theoretical DIC concentration was chosen in both cases to be greater than or equal to 3200 µmol/L, which triggered the precipitation of aragonite in the MAM experiment (Figure 4). The environment was sealed for the entire duration of these experiments. As shown in Figure 6, continuous decreases in DIC and TA are observed from the start of the MAC experiment, indicating continuous degassing and precipitation, consistent with the measured increase in CO_2_ concentration in the surrounding atmosphere (Figure 7). The results from the MN experiment in Figure 8 show a similar trend in DIC, while the measurements of TA are more erratic but stable, which may indicate degassing and, to a lesser extent, some precipitation.

## 3. Discussion

The experiments described in the previous section enable us to widen the perspective on the processes for treating seawater with buffered solutions enriched with CO_2_. The overall purpose of the experiments was to assess the efficiency of the alkalinity enhancement process (i.e., the fraction of CO_2_ actually introduced into seawater, mainly as bicarbonates) and its efficacy (i.e., the stability over time of the solutions, without precipitation of minerals or degassing of CO_2_). Here, we concentrate on the discussion of the SN experiments, which are based on the application of the revised BAWL technology implemented by Limenet^®^ on natural seawater.

The hypotheses underlying the BAWL technology that we wanted to test are:

- By injecting a CO_2_ solution pre-equilibrated at the same pH as natural seawater, one induces the least perturbation to the chemical equilibria of the carbonate system and to the natural environment. In particular, the pH should remain constant both after the initial treatment and over longer times;
- CO_2_ remains in the seawater solution mainly in the form of bicarbonate, so that the alkalinity and carbon content should increase, without precipitation of mineral phases or degassing of CO_2_;
- The efficiency is high, meaning that the measured increase of DIC matches the added quantity over a long time.

One major concern for marine sequestration approaches is that seawater is already oversaturated with calcium carbonates. Therefore, any further addition increases the risk of precipitation and degassing. All results indicate that there is indeed an upper limit, above which it is impossible to increase the carbon content of seawater. This affects the CO_2_ storage and the method efficiency, i.e., hypotheses (b) and (c). Below the critical concentration, all the previous interrelated hypotheses are simultaneously verified.

The natural seawater solutions treated with the Limenet^®^ process had a stable pH around the natural value of 8.1, up to DIC additions of 1500 µmol/L (Figure 1). Therefore, there are no special concerns about hypothesis (a). Also, the DIC and TA are stable when seawater is treated within this concentration limit, showing an average variation of 3 to 4%, the same as observed for untreated natural seawater. Statistical descriptors of the data are collected in Table A4 and Table A5 in Appendix A.

The DIC and TA drop by more than 60% when seawater is treated with the most concentrated solutions (see more details in Table A4, Table A5 and Table A6). The decrease in carbon content observed for DIC additions > 1500 µmol/L is probably due to a combination of CO_2_ degassing and precipitation of carbonate minerals. Once nucleation triggers the precipitation of carbonates, it can quickly proceed to significantly reduce Ω, in addition or in synergy with degasification.

The critical Ω of aragonite and calcite were recognized as important indicators of the likelihood of precipitation [7,8]. Marion et al. [35] suggested 18.8 and 12.3 for Ω_Ca_ and Ω_Ar_, respectively. In more specific experiments on OAE, Moras et al. [7] reported aragonite formation at much lower supersaturations and suggested a safe threshold of Ω_Ar_ = 5 to avoid “runaway” precipitation. In our SN1-1500 samples, there is no evidence of precipitation even if the Ω_Ar_ has an average value of 7.7. Such discrepancies among the defined thresholds may originate from several factors. First of all, we point out that the supersaturation states are not measured directly, but they are calculated by geochemical software that may apply different models. Secondly, one should take into account the specific technologies and chemicals used in the OAE operations, as well as the origin of the seawater (location, temperature, salinity, etc.). Finally, there are factors such as the presence of organic matter, pollutants, colloidal particles, and marine organisms that are not taken into account in the evaluation of Ω_Ar_, but they can certainly affect precipitation reactions [36,37,38]. For these reasons, we suggest the increase of Ω relative to that of the starting SW [rΩ, see Equation (5)] as a possible indicator for defining a safe OAE application.

Notwithstanding the different approaches to defining the threshold, when the limit is reached, the carbon storage efficiency drops significantly. The efficiency [η(t)] can be defined as the ratio between the observed increase in the carbon content of seawater and the theoretical one (Δ_DIC_). Our notation indicates that it is a time-dependent quantity.

Let DIC(*t*) and DIC_SW_(*t*) be the measured values of DIC for a given treatment and for untreated natural seawater, measured in the laboratory at the same time *t*. These two concentrations change over time, also due to processes that are unrelated to the loss of carbon, such as water evaporation and biological activity (the samples were kept in the lab at room temperature, in open glass bottles).

We obtain the efficiency as the product of two factors. The first ($\eta_{0}$) depends on phenomena occurring during the initial addition of carbon, the second one ($\eta_{St}$) during the subsequent stability tests:

(6) $$\eta \left(t\right)=\eta_{0}\times \eta_{St}\left(t\right).$$

These are given by:

(7) $$\eta_{0}=\frac{DIC\left(0\right)-DIC_{SW}\left(0\right)}{\Delta_{DIC}}$$

and:

(8) $$\eta_{St}(t)=\frac{1}{r(t)}\times \frac{DIC\left(t\right)-DIC_{SW}\left(t\right)}{DIC\left(0\right)-DIC_{SW}\left(0\right)}$$

where:

(9) $$r\left(t\right)=\frac{DIC_{SW}\left(t\right)}{DIC_{SW}\left(0\right)}$$

**Figure 9:**
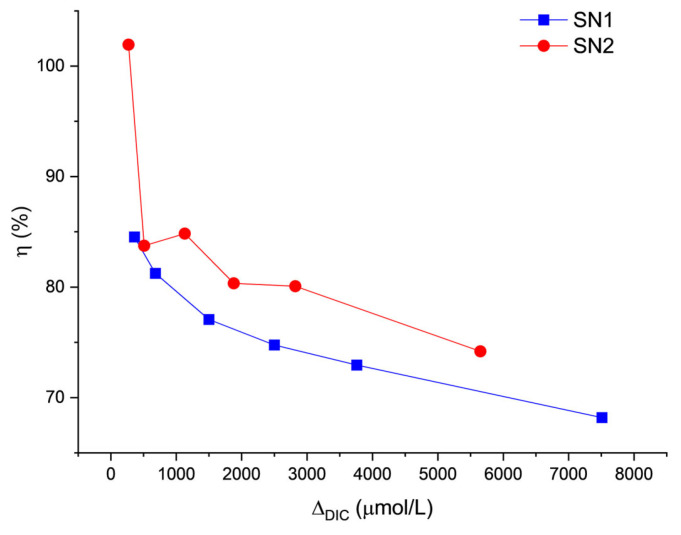
Process efficiency on day 1 as a function of DIC addition (Δ_DIC_).

The value of $\eta_{0}$ takes into account non-idealities that may occur in the reactor and in the line from the reactor to the delivery point, which reduce the amount of carbon taken up by the seawater solutions before discharge. Our estimates, based on the extrapolation of DIC data measured on day 1 (see Figure 9), lead to $\eta_{0}\approx \;$80% for $\Delta_{DIC}\geq 360$ μmol/L. This value could be increased by optimizing the process parameters. The efficiency of stability includes a correction factor r(t) that takes into account the already mentioned phenomena, which also occur in natural seawater under our laboratory conditions and affect all the DIC values, even though they are unrelated to the loss of carbon.

**Figure 10:**
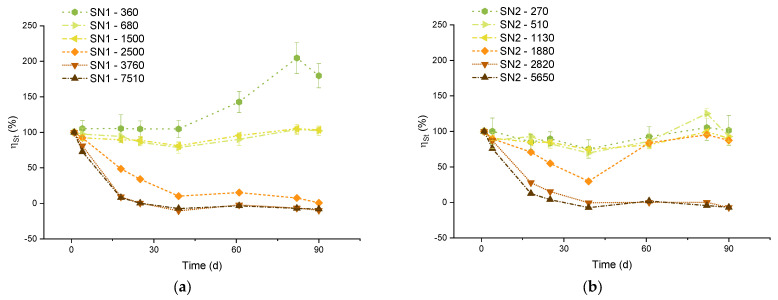
Stability efficiency over time. The efficiency of SN1 (**a**) and SN2 (**b**) samples are represented together. The name of the series represents the µmol/L of carbon theoretically added to the solution (Δ_DIC_).

Figure 10 shows the $\eta_{St}$ trends for samples with Δ_DIC_ higher than 270 µmol/L. The data for lower concentrations are not reported here because they are subject to very large errors. All samples with carbon addition between 510 and 1500 µmol/L share a similar trend: an average stability efficiency between 88% and 94% and a standard deviation of 8–9% (except for the 510 µmol/L theoretical DIC addition, for which the standard deviation was 16%). This implies an overall process efficiency of the order of 70%.

The sample with Δ_DIC_ equal to 360 µmol/L shows an efficiency that grows far above the 100% limit. This anomalous behavior is likely due to contamination of the sample after day 39, as it is not observed in all the other samples.

On the other hand, for the samples with Δ_DIC_ higher than 1500 µmol/L, the efficiency drops dramatically within a few days. For the highest concentrations, the efficiency is actually close to zero or even negative. A negative efficiency indicates a final DIC content lower than in untreated seawater. This agrees with the observed decrease of DIC in Figure 1 and the runaway precipitation of carbonate minerals, similar to the discussion by Moras et al. [7], Hartmann et al. [8], and Varliero et al. [39].

A final remark on efficiency is related to CO_2_ equilibrium with the atmosphere. Figure 1a,d shows a small increase in pH from day 1 to day 4 for all the samples, including seawater. This is likely due to the equilibration of the solution with the atmosphere by degassing. The importance of degassing is also highlighted by the experiments with small Δ_DIC_ in artificial seawater (SA and MAC, see Figure 3, Figure 6 and Figure 7). In those experiments, atmospheric CO_2_ increased without precipitation. Indeed, the pH of artificial seawater is generally lower than that of natural seawater, so it is understandable that degassing is more prominent.

## 4. Materials and Methods

### 4.1. Natural Seawater

Natural seawater has been used for two experimental configurations: single and multiple alkalinity dosages. Seawater has been sampled on two different occasions. Sampling for the SN1 and SN2 experiments occurred in September and October 2022 in La Spezia (Liguria, Italy) at the CSSN (Naval Support and Experimentation Center; coordinates: 44.095863, 9.862471). The MN experiment used water collected in February 2024 in La Spezia Bay (44.1013006, 9.8280323), and was stored in glass or polycarbonate Nalgene containers.

### 4.2. Artificial Seawater

Artificial seawater was prepared by dissolving NaCl, Na_2_SO_4_, KCl, MgCl_2_·6H_2_O, and CaCl_2_ salts in purified water, with the relative abundances proposed by Roy et al. [40] reported in Table 2. It was then stored in polycarbonate Nalgene tanks. All salts were Labkem (located in Barcelona, Spain) products, purchased from Labbox, and used without further purification.

### 4.3. Treatment with Ca(HCO_3_)_2_

**Figure 11:**
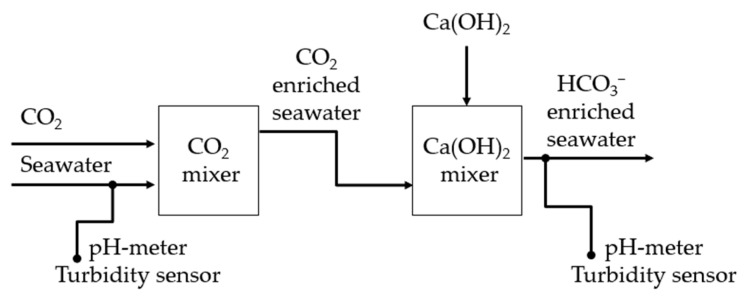
Scheme of the Limenet^®^ process applied to produce a high alkaline solution with natural seawater, and the sensors used to control the system.

Figure 11 shows a schematic block diagram of the Limenet^®^ system as implemented in La Spezia. Using a draft pump, about 25 L/s of seawater was collected at a depth of 2 m. After about 10 s, a gaseous stream of 100% CO_2_ was injected. After about 180 s, a slurry of Ca(OH)_2_ was dosed into the acidic stream of seawater and CO_2_ to reach the same pH as fresh seawater (i.e., about pH 8.1). The slurry was composed of 30 parts seawater and 1 part Ca(OH)_2_ by weight. The proportion of CO_2_ and seawater was controlled by a flux valve, while the amount of Ca(OH)_2_ was verified by weighing the hydroxide consumed. Table 3 summarizes the proportion of seawater, CO_2_, and Ca(OH)_2_.

pHSense 5–381 and TurbSense SN—TSIR—9667 probes were used to monitor pH and turbidity in the system. CO_2_ was provided by AirLiquide, while Ca(OH)_2_ powder was supplied by Unicalce.

The bicarbonate-enriched seawater exiting the plant was mixed with natural seawater to recreate different dilution ratios. Three sets of samples were produced: SN1 with a ratio of 3000 m^3^/ton between seawater and CO_2_; SN2 and SN3 with a ratio of 4000 m^3^/ton (see Table A6 for SN3). The bicarbonate-enriched solutions collected were diluted with fresh seawater, using variable proportions, namely 1:0, 1:1, 1:4, 1:10, 1:20, and 1:100 mass ratios between the alkaline solution and fresh seawater (see Table 4 for the corresponding Δ_DIC_).

After preparation, the containers were capped and transported on the same day to the laboratory of the Department of Chemistry, Politecnico di Milano, without any thermostatic storage device or other conditioning.

DIC and TA analysis was carried out within 24 h after collection. It was repeated once a week for one month and then twice a week for the last two months, for a total of 90 days.

Each sample was stored in two 500-mL borosilicate glass bottles and uncapped to allow them to reach equilibrium with CO_2_ under laboratory conditions. On day 1, pH and conductivity measurements were carried out to check the consistency between the two containers. We excluded the measurements on day 53 from Section 2 because the first bottles of each sample were almost empty and therefore more affected by evaporation. In Figure 1, for some solutions (especially SN2–1880), there is a visible gap between day 39 (last measurement from the first bottle) and day 61 (first measurement from the second bottle).

### 4.4. Treatment with NaHCO_3_

Experiments SA, MAM, MAC, and MN took place at the Politecnico di Milano. In these experiments, powdered sodium bicarbonate (NaHCO_3_) was added to 4.5 L of natural seawater.

NaHCO_3_ was a Labkem product from Labbox, and used without further purification.

In the SA experiments, sodium bicarbonate was added in a single dosage (2.0, 2.4, and 2.8 mmol/L). 2 mmol/L is the value of NaHCO_3_ suggested by Millero [31] for artificial seawater to mimic the natural seawater pH and alkalinity. These experiments were repeated twice. Furthermore, a control test, without NaHCO_3_ addition, was conducted.

After NaHCO_3_ addition, the beaker was confined inside a sealed poly(methyl methacrylate) plexiglass cabinet with a volume of 0.335 m^3^ to avoid exchanges of air with the external environment of the laboratory, having an air/water volumetric ratio of 74.3. The windows were opened approximately two hours before the analysis to maintain the concentration of CO_2_ similar among different experiments and to allow CO_2_ equilibration.

Probes were placed inside the cabinet to continuously measure pH, conductivity, and temperature of the artificial seawater. A CO_2_ sensor was used to measure its concentration (in ppm) in the atmosphere inside the cabinet. DIC and alkalinity were analyzed before and immediately after the NaHCO_3_ addition. At the end of the experiment, i.e., after about 48–72 h, the cabinet was opened, and all measurements were repeated.

The MAM experiments were performed in artificial seawater. Sodium bicarbonate was dosed in multiple stages, opening the cabinet for dosages and samplings. The addition was done step by step over 24 days, from 2000 to 5200 µmol/L.

The MN and MAC experiments were performed with 4 L of solution instead of 4.5 L, thus with an air/water volumetric ratio of 83.35. The cabinet was closed for the entire duration of the experiments, and sample suction and alkalinity injection were done through a 150 mL syringe by piping from the inside to the outside of the cabinet and controlled by a manually driven valve. NaHCO_3_ was pre-dissolved in a treated solution sampled by the syringe and then re-injected into the solution. To maintain the volume of the solution, treated seawater samples were kept outside the cabinet and added to replace the seawater sampled for measuring the DIC and TA. Before the first injection, the artificial and natural seawater were equilibrated with air inside the closed cabinet for three and one day, respectively. TA, DIC, pH, and conductivity were measured by periodic sampling, and CO_2_ concentration in the air was continuously recorded.

For all experiments, temperature was not controlled; the maximum and minimum values recorded during the entire duration of the experiments were about 21 and 16 °C, respectively.

### 4.5. Measurements

Before the first measurements of a new bottle, each sample was vacuum filtrated with sieves of 2–3 μm cut-off to remove large particles that could affect the subsequent analyses. Moreover, filtration allows for the identification of the precipitates’ nature and composition by X-ray diffraction analysis (XRD) using a Rigaku-Synergy-S single-crystal diffractometer. This equipment was necessary given the small amount of precipitate that did not allow a classical powder XRD measurement.

pH and conductivity were measured using electrode sensors from MATTLER TOLEDO Seven excellence. The pH probe was calibrated every two weeks according to the NIST scale; then, the values were corrected on the total scale, as suggested by Badocco et al. [41].

Total alkalinity was measured by automatic titration (Hanna Instruments HI84531). The pH probe was calibrated every two weeks while the pumping system was calibrated every day.

Dissolved inorganic carbon was measured by acidification and non-dispersive infrared absorbance (Analytik Jena multi NC 2100S). The machine calculates DIC concentration as the average of three measurements. If the average has a variation coefficient higher than 2%, a fourth measurement is provided, and one is discarded. We verified the calibration by measuring a 2500 µmol/L standard.

Atmospheric CO_2_ was measured using a sensor (ITSENSOR RCO2-W) located inside the cabinet.

### 4.6. Speciation and Phase Equilibria Simulation

The supersaturation Ω of aragonite has been calculated with the CO2SYS Excel Macro version 2.5 [42], using salinity, temperature, DIC, and pH as input data to characterize the carbonate system. The software was set on the pH total scale, using constants from Mehrbach [43] refit by Dickson and Millero [44] for the carbonate system, Dickson [45] for KHSO_4_, and Uppström [46] for B_T_. Practical salinity was calculated from the measured conductivity [47]. The calculation of Ω in CO2SYS does not consider the variation of Ca^2+^ due to the dissolution of Ca(OH)_2_ and precipitation of CaCO_3_, so the value was corrected as suggested by Moras et al. [7].

For the experiments in artificial seawater, a set of simulations was performed to determine the concentrations of NaHCO_3_. The aim was to ensure that Ω of aragonite did not exceed 5, i.e., the threshold value above which seawater is so oversaturated as to cause the precipitation of carbonates and the consequent release of CO_2_ into the atmosphere [8]. These simulations were performed with PHREEQC software version 3.7.0 [48], with the dataset “phreeqc.dat”.

## 5. Conclusions

We have presented a series of experiments on bicarbonate-enriched seawater, including both natural and artificial variants. The aim was to assess the factors affecting the stability and overall efficiency of the storage process, against adverse mechanisms such as CO_2_ degassing and precipitation of carbonate minerals [see e.g., Equation (3)].

The experiments on natural seawater presented in this work enable us to conclude that, for carbon additions up to 1500 µmol/L, the carbonate system and the carbon storage efficiency are stable over time. Mixing seawater with calcium bicarbonate solutions prepared with the Limenet^®^ process results in stable preservation of CO_2_ for over three months. Notably, the duration of these experiments is almost unprecedented for this kind of study. On the other hand, higher concentrations (with total DIC of ca. 4100 µmol/L, equivalent to a carbon addition of about 1800 µmol/L) may lead to precipitation and loss of efficiency. Experiments on artificial seawater, treated with solid NaHCO_3_, show precipitation and degassing for an increase in carbon content of ca. 1200 µmol/L, corresponding to a total DIC of 3200 µmol/L.

Considering the uncertainties of our measurements and environmental variance, we may conclude that a safe limit for the increase in carbon content in our seawater samples is about 1000 µmol/L. It is also important to consider that the precipitation observed above this threshold occurs only after several days. In a real-world application in a marine environment, this delay is likely sufficient to achieve significant dilution and avoid this pitfall, even for higher DIC additions.

## Figures and Tables

**Table 1:** Series of seawater samples and experiments. Each row represents a set of experiments conducted with different DIC additions.

Code	Mode	Seawater	Environment	Treatment	MaxΔ_DIC_ (µmol/L)	Initial DIC (µmol/L)	Duration (Days)
SN1	Single	Natural	Open	Ca(HCO_3_)_2_	7510	2370	90
SN2	Single	Natural	Open	Ca(HCO_3_)_2_	5650	2370	90
SA	Single	Artificial	Closed	NaHCO_3_	800	2000	3
MAM	Multiple	Artificial	Mixed	NaHCO_3_	3200	2000	24
MAC	Multiple	Artificial	Closed	NaHCO_3_	400	2800	16
MN	Multiple	Natural	Closed	NaHCO_3_	1000	2470	52

**Table 2:** Concentration of salts in artificial seawater [40]. Amount expressed as grams in each liter of distilled water added.

Salts	Concentration (g/L)
NaCl	25.14
Na_2_SO_4_	4.18
KCl	0.79
MgCl_2_·6H_2_O	11.19
CaCl_2_	1.20

**Table 3:** Seawater and calcium hydroxide used to produce samples SN1 and SN2.

	SN1	SN2
Seawater (m^3^)	3000	4000
Ca(OH)_2_ (ton)	0.874	0.874
CO_2_ (ton)	1.000	1.000

**Table 4:** Conversion from dilution ratios to dissolved inorganic carbon added initially to the solution (Δ_DIC_).

Dilution Ratio	SN1 Δ_DIC_ (µmol/L)	SN2 Δ_DIC_ (µmol/L)
SW	0	0
1:0	7510	5650
1:1	3760	2820
1:2	2500	1880
1:4	1500	1130
1:10	680	510
1:20	360	270
1:100	70	60

## Data Availability

One Excel file containing the data related to this study is included within the supporting information.

## Supplementary Materials

The following supporting information can be downloaded at: https://www.mdpi.com/article/10.3390/molecules29174069/s1. The Supporting Material containing the data related to this study is available as a zipped Excel File.

## Appendix A

**Table A1:** Saturation state (Ω) of aragonite for SN1 set of samples over time.

Time (d)	SW	SN1-7510	SN1-3760	SN1-2500	SN1-1500	SN1-680	SN1-360	SN1-70
1	4.21	8.94	9.91	8.11	6.46	5.12	4.60	4.25
4	4.30	8.21	9.67	10.20	7.73	5.82	5.08	4.53
18	4.03	4.25	4.22	6.33	7.61	5.41	5.01	4.10
25	4.20	3.82	3.96	5.80	8.05	6.03	5.09	4.51
39	3.69	2.33	2.81	4.49	7.72	5.65	4.81	4.34
61	3.34	2.65	3.05	3.95	7.14	5.01	4.76	3.99
82	3.69	2.48	3.05	3.53	7.88	5.68	5.37	4.81
90	4.60	2.99	3.44	4.26	9.18	6.50	6.68	5.57

**Table A2:** Saturation state (Ω) of aragonite for SN2 set of samples over time.

Time (d)	SW	SN2-5650	SN2-2820	SN2-1880	SN2-1130	SN2-510	SN2-270	SN2-60
1	4.21	7.88	8.21	6.83	5.93	4.92	4.61	4.42
4	4.30	8.20	10.39	8.79	7.11	5.45	5.03	4.58
18	4.03	4.75	5.45	7.53	6.64	5.01	4.36	4.07
25	4.20	4.07	4.99	7.17	7.45	5.58	4.97	4.50
39	3.69	3.03	3.46	5.58	6.08	4.84	4.78	4.23
61	3.34	3.09	3.19	7.72	5.48	4.31	3.99	3.50
82	3.69	3.48	3.73	9.76	7.09	5.35	4.69	4.32
90	4.60	3.37	3.80	10.39	8.00	6.12	5.71	5.10

**Table A3:** Data from SA experiments. Each row refers to a single experiment. Δ_DIC_ is the addition of NaHCO_3_. pH_f_ is the final pH. (TA_f_−TA_i_) and (DIC_f_−DIC_i_) are the differences between the final values of TA and DIC and those referred to measurements taken just after the addition of bicarbonate. CO_2,f_−CO_2,i_ indicates the variation of CO_2_ in the gas phase, expressed as µmol of gaseous CO_2_ per L of solution volume. Δ_C,tot_ is the total variation of carbon in the system, considering the atmosphere and the solution contribution. Ω_Ar_ is the calculated aragonite saturation state, at the beginning of each experiment, right after the bicarbonate addition.

Δ_DIC_ (µmol/L)	pH_f_	TA_f_−TA_i_ (µeq/L)	CO_2,f_−-CO_2,I_ (µmol/L)	DIC_f_−-DIC_i_ (µmol/L)	Δ_C,tot_ (µmol/L)	Ω_Ar_
0	8.03	18	70	−92	−22	2.58
0	7.93	68	57	−94	−37	2.28
400	7.98	116	105	−122	−17	2.96
400	7.96	−16	149	−129	20	2.53
800	8.00	112	66	−169	−103	3.37
800	8.01	−4	66	−143	−77	3.71

**Table A4:** Average, standard deviation, and averaged percentage variation (AV%) for SN1 of DIC and TA during the three months of sample analysis.

Δ_DIC_	DIC (µmol/L)	AV%	TA (µmol/L)	AV%
0	2483 ± 87	4%	2661 ± 92	3%
7510	3294 ± 2035	62%	3373 ± 1950	58%
3760	2935 ± 1106	38%	3079 ± 1120	36%
2500	3135 ± 644	21%	3348 ± 652	19%
1500	3633 ± 169	5%	3910 ± 199	5%
680	3036 ± 116	4%	3237 ± 193	6%
360	2915 ± 206	7%	3085 ± 239	8%
70	2705 ± 199	7%	2883 ± 241	8%

**Table A5:** Average, standard deviation, and averaged percentage variation (AV%) for SN2 of DIC and TA during the three months of sample analysis.

Δ_DIC_	DIC (µmol/L)	AV%	TA (µmol/L)	AV%
0	2483 ± 87	4%	2661 ± 92	3%
5650	3245 ± 1637	50%	3371 ± 1552	46%
2820	3021 ± 864	29%	3131 ± 928	30%
1880	3576 ± 471	13%	3842 ± 434	11%
1130	3358 ± 109	3%	3547 ± 155	4%
510	2888 ± 108	4%	3030 ± 95	3%
270	2751 ± 97	4%	2844 ± 106	4%
60	2594 ± 99	4%	2694 ± 118	4%

**Table A6:** Average, standard deviation, and averaged percentage variation (AV%) for SN3 of DIC and TA during the three months of sample analysis.

Δ_DIC_	DIC (µmol/L)	AV%	TA (µmol/L)	AV%
0	2472 ± 77	3%	2686 ± 102	4%
2820	2645 ± 1003	38%	2789 ± 1081	39%
1130	3417 ± 110	3%	3758 ± 226	6%
510	2904 ± 94	3%	3134 ± 148	5%
270	2773 ± 88	3%	2936 ± 175	6%
60	2556 ± 91	4%	2756 ± 121	4%

## References

[1] Intergovernmental Panel on Climate Change (IPCC) Technical Summary (2023). Climate Change 2022—Mitigation of Climate Change: Working Group III Contribution to the Sixth Assessment Report of the Intergovernmental Panel on Climate Change.

[2] Celia M.A., Bachu S., Nordbotten J.M., Bandilla K.W. (2015). Status of CO_2_ Storage in Deep Saline Aquifers with Emphasis on Modeling Approaches and Practical Simulations. Water Resour. Res..

[3] Wang N., Akimoto K., Nemet G.F. (2021). What Went Wrong? Learning from Three Decades of Carbon Capture, Utilization and Sequestration (CCUS) Pilot and Demonstration Projects. Energy Policy.

[4] Lane J., Greig C., Garnett A. (2021). Uncertain Storage Prospects Create a Conundrum for Carbon Capture and Storage Ambitions. Nat. Clim. Chang..

[5] Global CCS Institute (2023). Global Status of CCS 2023.

[6] Rau G.H., Caldeira K. (1999). Enhanced Carbonate Dissolution: A means of sequestering waste CO_2_ as ocean bicarbonate. Energy Convers. Manag..

[7] Moras C.A., Bach L.T., Cyronak T., Joannes-Boyau R., Schulz K.G. (2022). Ocean Alkalinity Enhancement—Avoiding Runaway CaCO3 Precipitation during Quick and Hydrated Lime Dissolution. Biogeosciences.

[8] Hartmann J., Suitner N., Lim C., Schneider J., Marín-Samper L., Arístegui J., Renforth P., Taucher J., Riebesell U. (2023). Stability of Alkalinity in Ocean Alkalinity Enhancement (OAE) Approaches—Consequences for Durability of CO_2_ Storage. Biogeosciences.

[9] Caserini S., Cappello G., Righi D., Raos G., Campo F., De Marco S., Renforth P., Varliero S., Grosso M. (2021). Buffered Accelerated Weathering of Limestone for Storing CO_2_: Chemical Background. Int. J. Greenh. Gas Control.

[10] Ringham M., Hirtle N., Shaw C., Lu X., Herndon J., Carter B., Eisaman M. (2024). A Comprehensive Assessment of Electrochemical Ocean Alkalinity Enhancement in Seawater: Kinetics, Efficiency, and Precipitation Thresholds. EGUsphere.

[11] Zeebe R.E., Wolf-Gladrow D.A. (2001). CO_2_ in Seawater: Equilibrium, Kinetics, Isotopes.

[12] Renforth P., Henderson G. (2017). Assessing Ocean Alkalinity for Carbon Sequestration. Rev. Geophys..

[13] Middelburg J.J., Soetaert K., Hagens M. (2020). Ocean Alkalinity, Buffering and Biogeochemical Processes. Rev. Geophys..

[14] Eisaman M.D., Geilert S., Renforth P., Bastianini L., Campbell J., Dale A.W., Foteinis S., Grasse P., Hawrot O., Löscher C.R. (2023). Assessing the Technical Aspects of Ocean-Alkalinity-Enhancement Approaches. State Planet.

[15] Caldeira K., Rau G.H. (2000). Accelerating Carbonate Dissolution to Sequester Carbon Dioxide in the Ocean: Geochemical Implications. Geophys. Res. Lett..

[16] Rau G.H. (2011). CO_2_ Mitigation via Capture and Chemical Conversion in Seawater. Environ. Sci. Technol..

[17] Chou W.-C., Gong G.-C., Hsieh P.-S., Chang M.-H., Chen H.-Y., Yang C.-Y., Syu R.-W. (2015). Potential Impacts of Effluent from Accelerated Weathering of Limestone on Seawater Carbon Chemistry: A Case Study for the Hoping Power Plant in Northeastern Taiwan. Mar. Chem..

[18] Kirchner J.S., Berry A., Ohnemüller F., Schnetger B., Erich E., Brumsack H.-J., Lettmann K.A. (2020). Reducing CO_2_ Emissions of a Coal-Fired Power Plant via Accelerated Weathering of Limestone: Carbon Capture Efficiency and Environmental Safety. Environ. Sci. Technol..

[19] Kirchner J.S., Lettmann K.A., Schnetger B., Wolff J.-O., Brumsack H.-J. (2020). Carbon Capture via Accelerated Weathering of Limestone: Modeling Local Impacts on the Carbonate Chemistry of the Southern North Sea. Int. J. Greenh. Gas Control.

[20] De Marco S., Varliero S., Caserini S., Cappello G., Raos G., Campo F., Grosso M. (2023). Techno-Economic Evaluation of Buffered Accelerated Weathering of Limestone as a CO_2_ Capture and Storage Option. Mitig. Adapt. Strateg. Glob. Chang..

[21] Limenet^®^, United States Patent and Trademark—Office (2024). Apparatus and Method for Accelerated Dissolution of Carbonates with Buffered. European Patent.

[22] Gattuso J.-P., Hansson L. (2011). Acidification: Background and History.

[23] Camatti E., Valsecchi S., Caserini S., Barbaccia E., Santinelli C., Basso D., Azzellino A. (2024). Short-Term Impact Assessment of Ocean Liming: A Copepod Exposure Test. Mar. Pollut. Bull..

[24] Fakhraee M., Li Z., Planavsky N.J., Reinhard C.T. (2023). A Biogeochemical Model of Mineral-Based Ocean Alkalinity Enhancement: Impacts on the Biological Pump and Ocean Carbon Uptake. Environ. Res. Lett..

[25] Schwinger J., Bourgeois T., Rickels W. (2024). On the Emission-Path Dependency of the Efficiency of Ocean Alkalinity Enhancement. Environ. Res. Lett..

[26] Paul A.J., Haunost M., Goldenberg S.U., Hartmann J., Sánchez N., Schneider J., Suitner N., Riebesell U. (2024). Ocean Alkalinity Enhancement in an Open Ocean Ecosystem: Biogeochemical Responses and Carbon Storage Durability. EGUsphere.

[27] Álvarez M., Sanleón-Bartolomé H., Tanhua T., Mintrop L., Luchetta A., Cantoni C., Schroeder K., Civitarese G. (2014). The CO_2_ System in the Mediterranean Sea: A Basin Wide Perspective. Ocean. Sci..

[28] Suitner N., Faucher G., Lim C., Schneider J., Moras C.A., Riebesell U., Hartmann J. (2024). Ocean Alkalinity Enhancement Approaches and the Predictability of Runaway Precipitation Processes—Results of an Experimental Study to Determine Critical Alkalinity Ranges for Safe and Sustainable Application Scenarios. EGUsphere.

[29] Schulz K.G., Bach L.T., Dickson A.G. (2023). Seawater Carbonate Chemistry Considerations for Ocean Alkalinity Enhancement Research: Theory, Measurements, and Calculations.

[30] Zhang Z., Xie Y., Xu X., Pan H., Tang R. (2012). Transformation of Amorphous Calcium Carbonate into Aragonite. J. Cryst. Growth.

[31] Millero F.J. (2007). The Marine Inorganic Carbon Cycle. Chem. Rev..

[32] https://www.eea.europa.eu/en/analysis/indicators/ocean-acidification#:~:text=Seawater%20pH%20has%20decreased%20from,modifying%20ecosystem%20services%20like%20fisheries.

[33] Intergovernmental Panel on Climate Change (IPCC) Technical Summary (2023). Climate Change 2021—The Physical Science Basis: Working Group I Contribution to the Sixth Assessment Report of the Intergovernmental Panel on Climate Change.

[34] Morse J.W., Arvidson R.S., Lüttge A. (2007). Calcium Carbonate Formation and Dissolution. Chem. Rev..

[35] Marion G.M., Millero F.J., Feistel R. (2009). Precipitation of Solid Phase Calcium Carbonates and Their Effect on Application of Seawater S A-T-P Models. Ocean. Sci..

[36] Jiang L., Feely R.A., Carter B.R., Greeley D.J., Gledhill D.K., Arzayus K.M. (2015). Climatological Distribution of Aragonite Saturation State in the Global Oceans. Glob. Biogeochem. Cycles.

[37] Zhang D., Lin Q., Xue N., Zhu P., Wang Z., Wang W., Ji Q., Dong L., Yan K., Wu J. (2019). The Kinetics, Thermodynamics and Mineral Crystallography of CaCO_3_ Precipitation by Dissolved Organic Matter and Salinity. Sci. Total Environ..

[38] Cyronak T., Schulz K.G., Jokiel P.L. (2016). The Omega Myth: What Really Drives Lower Calcification Rates in an Acidifying Ocean. ICES. J. Mar. Sci..

[39] Varliero S., Buono A., Caserini S., Raos G., Macchi P. (2024). Chemical Aspect of Ocean Liming for CO_2_ Removal: Dissolution Kinetics of Calcium Hydroxide in Seawater. ACS Eng. Au.

[40] Roy R.N., Roy L.N., Vogel K.M., Porter-Moore C., Pearson T., Good C.E., Millero F.J., Campbell D.M. (1993). The Dissociation Constants of Carbonic Acid in Seawater at Salinities 5 to 45 and Temperatures 0 to 45 °C. Mar. Chem..

[41] Badocco D., Pedrini F., Pastore A., di Marco V., Marin M.G., Bogialli S., Roverso M., Pastore P. (2021). Use of a Simple Empirical Model for the Accurate Conversion of the Seawater PH Value Measured with NIST Calibration into Seawater PH Scales. Talanta.

[42] Lewis E., Wallace D. (2011). MS Excel Program Developed for CO_2_ System Calculations.

[43] Mehrbach C., Culberson C.H., Hawley J.E., Pytkowicx R.M. (1973). Measurement of the Apparent Dissociation Constants of Carbonic Acid in Seawater at Atmospheric Pressure. Limnol. Oceanogr..

[44] Dickson A.G., Millero F.J. (1987). A Comparison of the Equilibrium Constants for the Dissociation of Carbonic Acid in Seawater Media. Deep Sea Research Part A. Oceanogr. Res. Pap..

[45] Dickson A.G. (1990). Standard Potential of the Reaction: AgCl(s) + 12H2(g) = Ag(s) + HCl(Aq), and the Standard Acidity Constant of the Ion HSO_4_^−^ in Synthetic Sea Water from 273.15 to 318.15 K. J. Chem. Thermodyn..

[46] Uppström L.R. (1974). The Boron/Chlorinity Ratio of Deep-Sea Water from the Pacific Ocean. Deep. Sea Res. Oceanogr. Abstr..

[47] Lewis E.L., Perkin R.G. (1981). The Practical Salinity Scale 1978: Conversion of Existing Data. Deep Sea Research Part A. Oceanogr. Res. Pap..

[48] Parkhurst D., Appelo C. (2013). Description of Input and Examples for PHREEQC Version 3: A Computer Program for Speciation, Batch-Reaction, One-Dimensional Transport, and Inverse Geochemical Calculations. US Geol. Surv. Tech. Methods.

